# A Novel Antigenic Site Spanning Domains I and III of the Zika Virus Envelope Glycoprotein Is the Target of Strongly Neutralizing Human Monoclonal Antibodies

**DOI:** 10.1128/JVI.02423-20

**Published:** 2021-04-12

**Authors:** Stephen D. Graham, Huy A. Tu, Benjamin D. McElvany, Nancy R. Graham, Ariadna Grinyo, Edgar Davidson, Benjamin J. Doranz, Sean A. Diehl, Aravinda M. de Silva, Alena Janda Markmann

**Affiliations:** a Department of Microbiology and Immunology, University of North Carolina at Chapel Hill, Chapel Hill, North Carolina, USA; b Department of Microbiology and Molecular Genetics, Vaccine Testing Center, Cellular, Molecular, and Biomedical Sciences Program, Larner College of Medicine, University of Vermont, Burlington, Vermont, USA; c Integral Molecular, Inc., Philadelphia, Pennsylvania, USA; d Department of Medicine, Division of Infectious Disease, University of North Carolina at Chapel Hill, Chapel Hill, North Carolina, USA; St. Jude Children’s Research Hospital

**Keywords:** B-cell responses, epitope, Zika virus, immune memory, immunology, monoclonal antibodies

## Abstract

Zika virus (ZIKV), a mosquito-transmitted flavivirus, caused a large epidemic in Latin America between 2015 and 2017. Effective ZIKV vaccines and treatments are urgently needed to prevent future epidemics and severe disease sequelae. People infected with ZIKV develop strongly neutralizing antibodies linked to viral clearance and durable protective immunity. To understand the mechanisms of protective immunity and to support the development of ZIKV vaccines, we characterize here a strongly neutralizing antibody, B11F, isolated from a patient who recovered from ZIKV. Our results indicate that B11F targets a complex epitope on the virus that spans domains I and III of the envelope glycoprotein. While previous studies point to quaternary epitopes centered on domain II of the ZIKV E glycoprotein as targets of strongly neutralizing and protective human antibodies, we uncover a new site spanning domains I and III as a target of strongly neutralizing human antibodies.

**IMPORTANCE** People infected with Zika virus develop durable neutralizing antibodies that prevent repeat infections. In the current study, we characterize a ZIKV-neutralizing human monoclonal antibody isolated from a patient after recovery. Our studies establish a novel site on the viral envelope that is targeted by human neutralizing antibodies. Our results are relevant to understanding how antibodies block infection and to guiding the design and evaluation of candidate vaccines.

## Abstract (precis)

People infected with Zika virus develop durable neutralizing antibodies that prevent repeat infections. In the current study, we characterize a ZIKV-neutralizing human monoclonal antibody isolated from a patient after recovery. Our studies establish a novel site on the viral envelope that is targeted by human neutralizing antibodies. Our results are relevant to understanding how antibodies block infection and to guiding the design and evaluation of candidate vaccines.

## INTRODUCTION

Zika virus (ZIKV) is a mosquito-borne flavivirus responsible for recent large epidemics accompanied by severe clinical manifestations such as Guillain-Barré syndrome and congenital birth defects (1). The ZIKV epidemic in South America in 2015 highlighted the need to understand the mechanisms of protective immunity in order to guide the development of vaccines and other countermeasures (2). Among flaviviruses, ZIKV is most closely related to the four dengue viruses (DENV-1 to DENV-4). Recent setbacks faced by DENV vaccine developers highlight the importance of a deeper understanding of immune-protective antigenic targets for pathogenic flaviviruses (3, 4).

In humans, infection with a single flavivirus is known to induce long-term, likely lifelong adaptive immune protection. An important component of the long-term protective immune response to flaviviral infections is the production of potently neutralizing antibodies (5). These durable protective antibodies generated after flaviviral infection are continuously secreted by long-lived plasma cells in the bone marrow and are produced during antigenic recall by memory B cells (MBCs) residing in lymphoid organs. Identifying the viral binding sites, or antigenic targets, of MBC-derived neutralizing antibodies helps us understand how the immune response prevents repeated infections by the same virus (6). Immunogenic epitopes can be used to directly inform vaccine design and also to aid in diagnostic design (7, 8).

The main target of human antibodies that neutralize flaviviruses is the envelope (E) glycoprotein, which covers the surface of the virion. Each E glycoprotein monomer contains three domains—E domain I (EDI), EDII, and EDIII—and a fusion loop at the tip of domain II (see Fig. 4) (9). E glycoproteins form stable homodimers, and 90 dimers assemble to form the outer envelope of the infectious virus. Primary flavivirus infections stimulate cross-reactive (CR) antibodies, which target epitopes conserved between closely related flaviviruses, as well as type-specific (TS) antibodies, which bind to unique epitopes on the infecting virus. CR antibodies do not reliably confer durable cross-protective immunity after a primary infection, most likely because they bind with low affinity to conserved epitopes that are not well exposed on the viral surface (10). In contrast, TS antibodies are often strongly neutralizing and are linked to long-term protection from reinfection by the same flavivirus (2).

Several groups have isolated a few strongly neutralizing human monoclonal antibodies (MAbs) from MBCs. The most potent antibodies have been mapped to complex epitopes centered on domain II of the E glycoprotein, with footprints that span two or more E glycoproteins (11–13). Recent studies have also identified a few strongly neutralizing MAbs for ZIKV that target epitopes in EDIII (14–17), although this domain does not appear to be a major target of polyclonal neutralizing antibodies in serum (7). Here, using MAbs isolated from individuals who have recovered from Zika virus infection, we define a novel antigenic site, between domains I and III of the ZIKV E glycoprotein, targeted by human antibodies that strongly neutralize ZIKV.

## RESULTS

### Isolation of a ZIKV type-specific and strongly neutralizing MAb, B11F.

**FIG 1:**
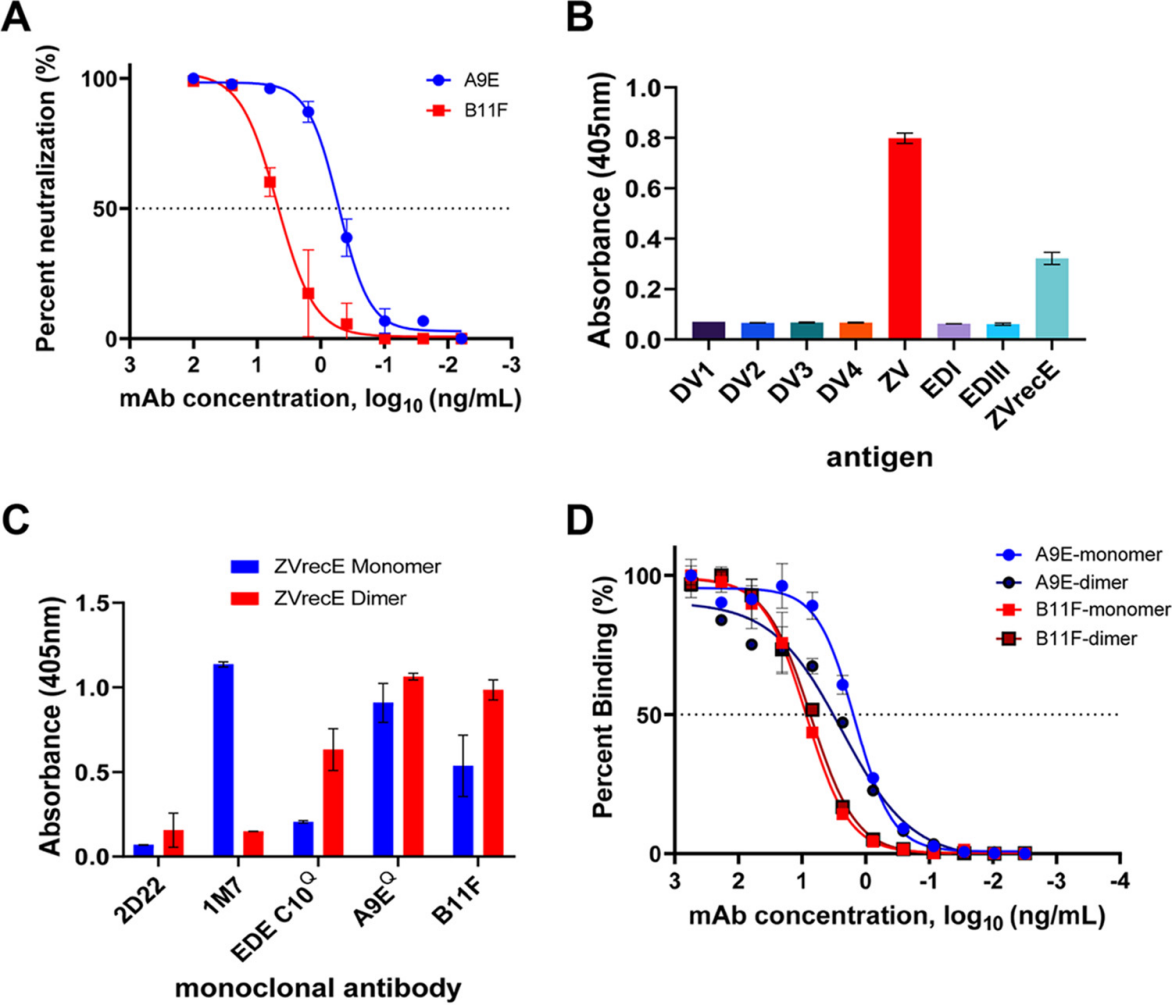
B11F binding specificity and virus neutralization as determined by ELISA. (A) ZIKV neutralization by B11F and A9E. Each value is the average for duplicate wells. Fifty percent neutralization occurred at concentrations of 3.22 ng/ml for B11F (squares) and 0.33 ng/ml for A9E (circles). The graph is representative of the results of three independent experiments. (B) ELISA for MAb B11F binding using whole virions, recombinant ZIKV E protein, EDI, and EDIII. DV, DENV; ZV, ZIKV. (C) B11F binding to ZIKV E protein monomers and dimers by capture ELISA. 2D22 is a DENV-2-specific MAb; 1M7 is a fusion loop-binding panflaviviral antibody; and EDE C10 binds a quaternary epitope present only on a dimeric antigen. For panels B and C, each value represents the average from duplicate wells, the background absorbance is 0.1 optical density unit, and the graph is representative of at least two independent experiments. (D) Binding of B11F and A9E to monomeric and dimeric forms of the ZIKV E protein. Blue circles, A9E and monomers (50% effective concentration [EC_50_], 1.7 ng/ml); black circles, A9E and dimers (EC_50_, 2.1 ng/ml); red squares, B11F and monomers (EC_50_, 7.6 ng/ml); black squares, B11F and dimers (EC_50_, 5.8 ng/ml). EC_50_ values are averages from two independent experiments. Each value is the average from duplicate wells.

Subject DT172 was a U.S. traveler who acquired a self-limited and uncomplicated ZIKV infection while traveling through Nicaragua and Colombia in 2015, early in the South American ZIKV epidemic. The subject had no neutralizing antibody titers to DENV-1 to -4 (<20) and a neutralization titer of 1:794 to ZIKV. As reported previously (11), when MBCs collected 3 months after recovery were immortalized by the 6XL method and tested, 0.9% of the cells were observed to be producing ZIKV-binding antibodies. To isolate ZIKV-neutralizing MAbs, we single-cell sorted MBCs and tested individual clones for ZIKV-binding and -neutralizing antibodies as described previously (11). We identified and sequenced a single IgG1 clone, named B11F, that strongly neutralized ZIKV (50% focus reduction neutralization titer [FRNT_50_], 3.22 ng/ml) (Fig. 1A). We were surprised to find that the sequence and gene usage of MAb B11F were similar to those of another human MAb, designated A9E, that we recently isolated from a different traveler who was infected with ZIKV in Brazil in 2017 (11).

Recombinantly produced B11F IgG1 bound to ZIKV but not to DENV-1 to -4 (Fig. 1B). The antibody also bound to the full-length ectodomain (recombinant ZIKV E [ZVrecE], wild-type protein) but not to domain I or III of ZIKV E glycoprotein (Fig. 1B). In solution at 37°C, the ZVrecE glycoprotein is in an equilibrium that greatly favors monomers over homodimers (9). We compared the binding of several ZIKV-specific human MAbs, including B11F, to ZVrecE monomers and stable homodimers (stabilized by the introduction of an intermolecular disulfide bond [18]). Control antibodies that preferentially bound monomers (panflaviviral fusion loop-targeting MAb 1M7) or homodimers (quaternary-epitope-specific MAb EDE C10) confirmed the oligomeric state of our antigens (Fig. 1C). B11F bound similarly to ZVrecE monomers and homodimers (Fig. 1D). We conclude that B11F is a potently neutralizing antibody that binds to a ZIKV type-specific epitope displayed on the ectodomain of ZIKV E glycoprotein but not on domains I and III alone.

### BOB assays with B11F and other well-characterized ZIKV-specific human MAbs.

**FIG 2:**
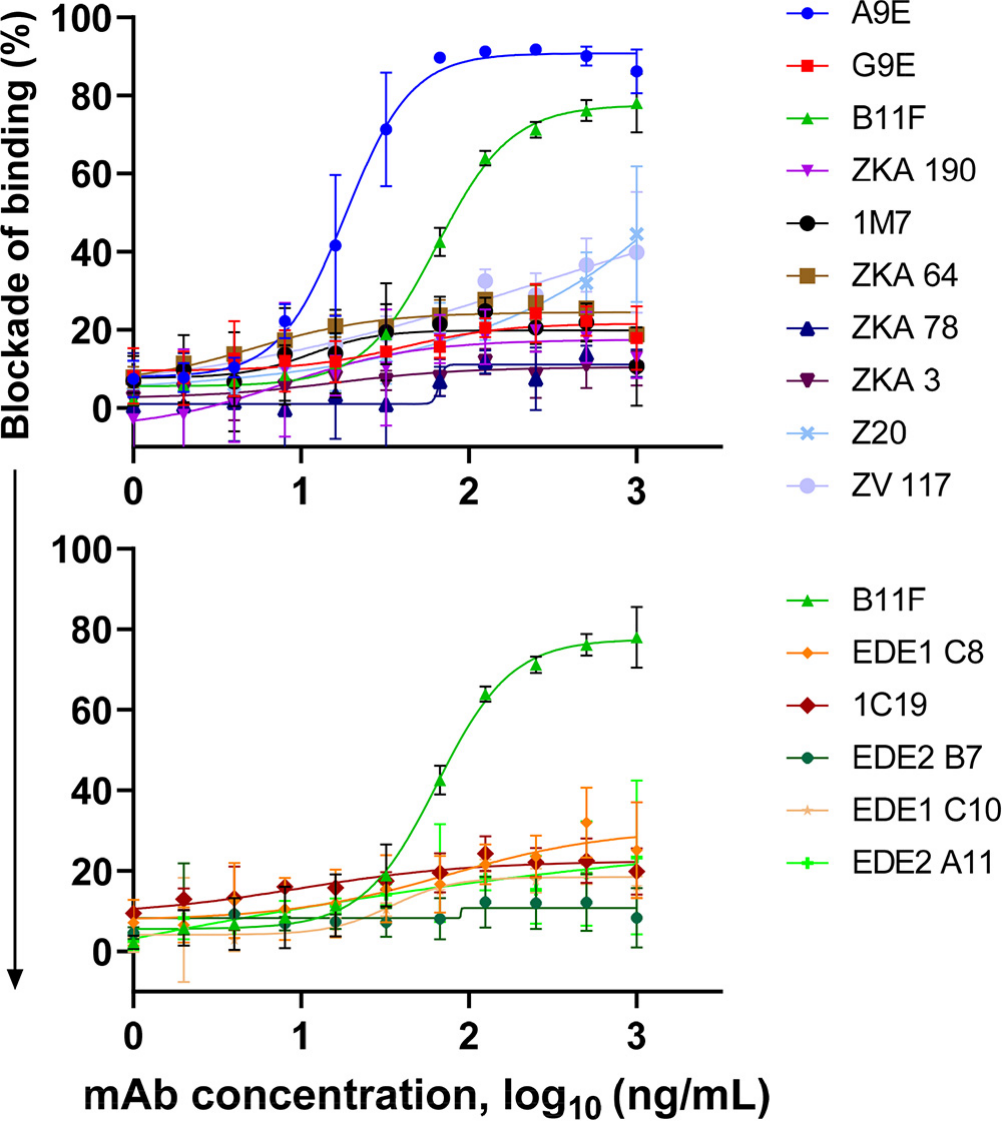
Zika virus blockade-of-binding ELISA results. (Top) B11F blockade with Zika virus-specific monoclonal antibodies. (Bottom) B11F blockade with dengue virus-specific monoclonal antibodies. Here, B11F is held at a constant concentration, and the *x* axis shows the varying concentrations of the competing monoclonal antibody. Error bars represent averaged data sets from two independent experiments.

To explore further how the ZIKV E glycoprotein binding site of B11F is related to known antibody epitopes on ZIKV, we performed antibody competition assays (blockade-of-binding [BOB] assays) with B11F against 14 ZIKV-specific MAbs or DENV and ZIKV cross-reactive MAbs with mapped epitopes. The EDE MAbs and 1C19 are ZIKV-binding and -neutralizing (except for EDE2 B7) MAbs that map to a region across EDII–EDIII (19–22). We compared the abilities of the different MAbs to block the binding of labeled B11F to intact ZIKV virions captured on an enzyme-linked immunosorbent assay (ELISA) plate (Fig. 2). Of the 14 antibodies tested, only MAb A9E blocked the binding of B11F to ZIKV. While the full epitope of A9E has not been mapped yet, our previous studies demonstrate that A9E targets an epitope centered on EDI that extends toward EDIII on a single E glycoprotein monomer (11). This supports the evidence that MAb B11F targets the EDI–EDIII region on the ZIKV E glycoprotein.

### Identification of ZIKV neutralization escape mutants.

**FIG 3:**
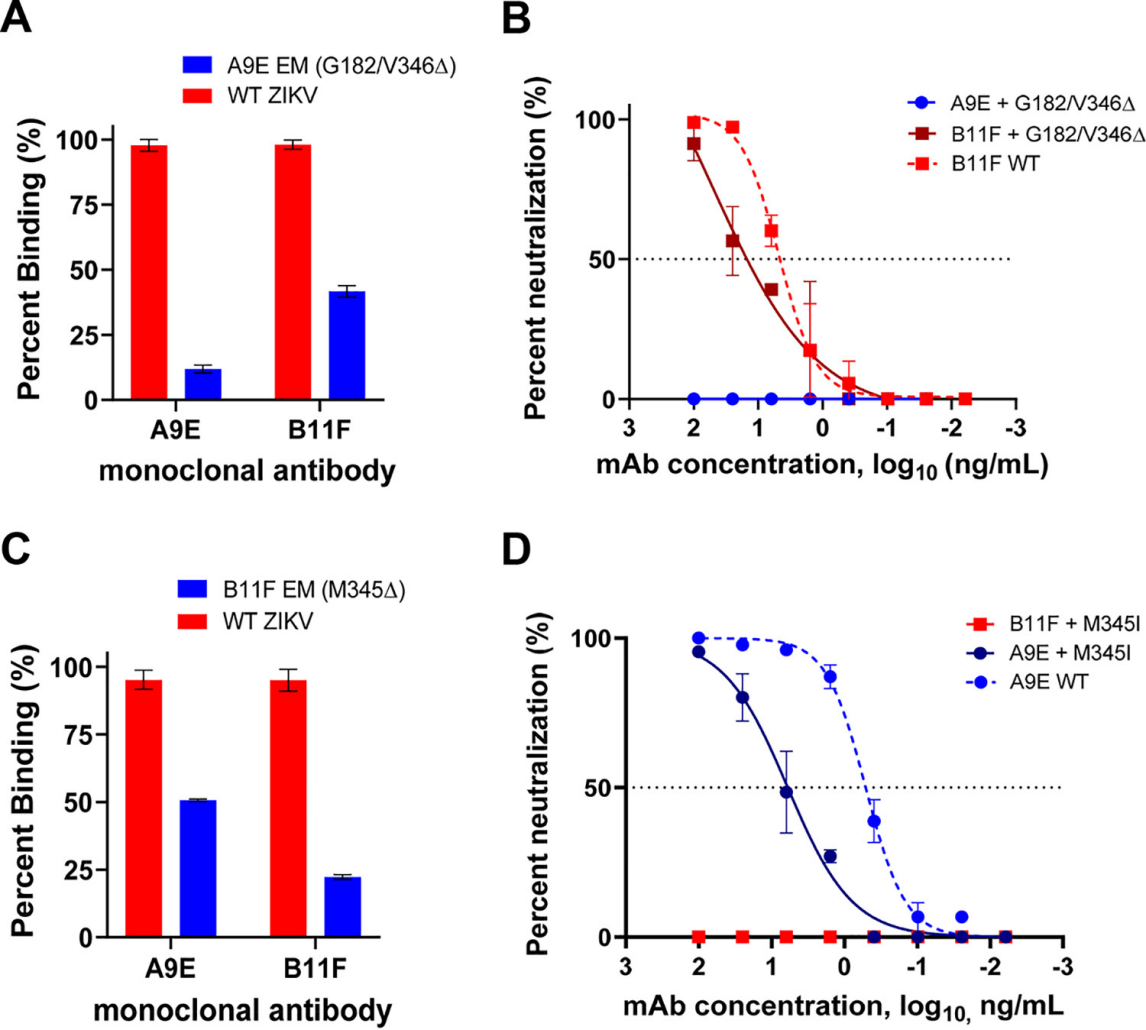
Binding and neutralization of escape mutant viruses. (A) Whole-virion capture ELISA binding results for the A9E escape mutant virus. Percentages of binding represent the average values for duplicate wells. (B) A9E escape mutant virus neutralization assay. Wild-type (WT) ZIKV was used as a positive control in all experiments. Mutations shown are those located on the A9E escape mutant virus. (C) Whole-virion capture ELISA binding results for the B11F escape mutant. (D) B11F escape mutant virus neutralization assay. Mutations shown are those located on the B11F escape mutant virus. All graphs shown are representative of at least two independent experiments.

**FIG 4:**
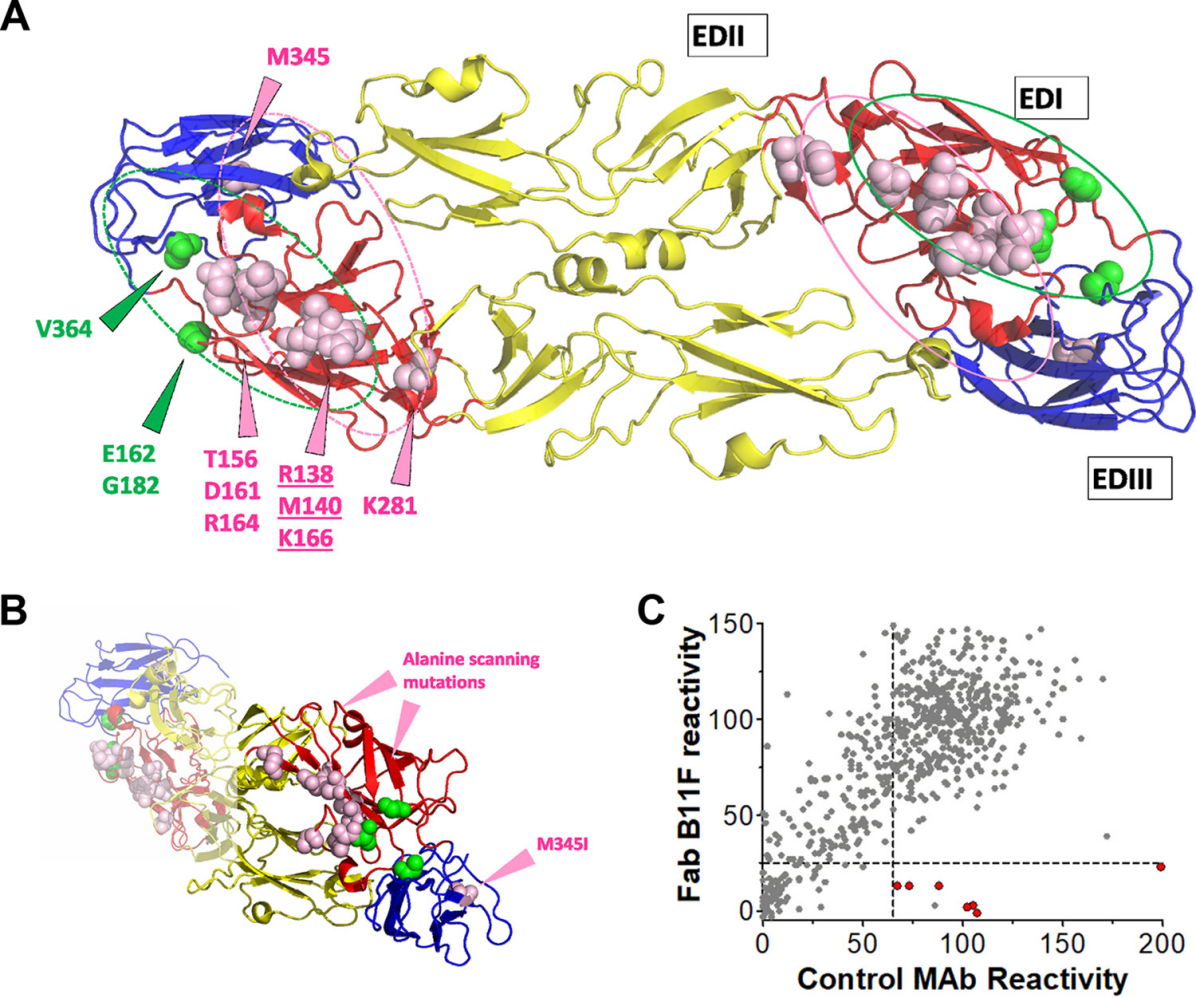
Epitope-mapping analysis of the B11F and A9E antibodies. (A) Zika virus envelope protein dimer (PDB code 5IRE) with domains labeled and color-coded. The locations of the B11F viral escape mutation (M345) (pink spheres within EDIII) and alanine-scanning mutations (pink spheres on EDI; underlined residues make the largest contributions to binding) and the locations of A9E escape mutations and alanine-scanning mutations (green spheres) are shown. The putative B11F MAb footprint is shown in pink, and the putative A9E MAb footprint in green. (B) Side/edge view displaying the distance between B11F escape mutation M345I and the B11F mutations identified by alanine scanning. The distance between the closest atoms of M345 and the alanine-scanning mutant residues for B11F (M345-N and D161-O) is approximately 27 Å. (C) Amino acid residues critical for B11F Fab binding to ZIKV envelope glycoprotein were determined by alanine-scanning shotgun mutagenesis. This plot shows the binding of B11F Fab to the mutants versus the binding of a set of control monoclonal antibodies. Red circles correspond to alanine mutants that reduce B11F Fab binding from that by control monoclonal antibodies.

The epitopes of MAbs that neutralize flaviviruses can be mapped by passaging the virus in the presence of the MAb under study to select for mutations that prevent antibody binding and neutralization. We reported previously on specific mutations in EDI (G182D) and EDIII (V364I) that led to escape from neutralization by MAb A9E (11) (see Fig. 4). Mutations that led to escape from binding and neutralization by MAb A9E moderately reduced the binding as well as the neutralization potency of B11F (FRNT_50_, ∼3 ng/ml [wild type] → ∼34 ng/ml [escape mutant]) (Fig. 3A and B). We also passaged ZIKV in the presence of MAb B11F and isolated an escape mutant virus that was able to replicate in the presence of B11F. The B11F escape mutant virus has a mutation to I at position M345, which is buried in the core of EDIII (Fig. 4A). The M345I mutation, which prevented binding and neutralization by MAb B11F, had a moderate effect on MAb A9E binding and led to a 10-fold reduction in A9E neutralization potency from that with the wild-type virus (A9E FRNT_50_, ∼0.3 ng/ml [wild type] → ∼6 ng/ml [escape mutant]) (Fig. 3C and D). These results are consistent with the notion that human MAbs B11F and A9E have overlapping but distinct epitopes.

### Alanine-scanning mutagenesis for epitope mapping.

We used a ZIKV prM/E glycoprotein expression library with single alanine mutations to identify mutations that reduced or eliminated MAb B11F Fab binding. This library and approach have been extensively validated for mapping ZIKV-binding antibodies (11, 13). Mutations at E glycoprotein residues Arg138, Thr156, Met140, Asp161, Arg164, Lys166, and Lys281 selectively reduced B11F binding while retaining the overall structural integrity of the glycoprotein (Fig. 4A). Mutations at residues M140, K166, and R138 had the largest impact on B11F binding and very little to no impact on MAb A9E (Table 1). All the residues identified by alanine-scanning mutagenesis are surface exposed on EDI. The shortest distance between the escape virus mutation M345I and an alanine scan-identified mutation, found between the N atom at M345 (M345-N) and D161-O, was a large distance of approximately 27 Å (Fig. 4B). Overall B11F Fab reactivity against the alanine mutagenesis library is shown in Fig. 4C, and the positions with the highest reactivities are shown in Table 1.

**TABLE 1:** Identification of residues critical for the binding of MAb B11F to ZIKV^*a*^

Mutation	Binding^*b*^ by MAb:	
	B11F	A9E
R138A	−1.4 (5.7)	78.2 (1.1)
K166A	2.4 (1.4)	93.4 (33.7)
M140A	3.1 (2.9)	107.6 (12.5)
R164A	12.7 (7.0)	105.2 (14.5)
D161A	13.3 (6.5)	40.5 (10.1)
K281A	13.5 (3.4)	77.1 (3.6)
T156A	23.4 (2.9)	59.7 (16.8)

a Shown are binding data for B11F and A9E with all ZIKV E protein clones identified as critical for B11F binding.

b Expressed as the mean percentage (range [expressed as half of the maximum value minus the minimum value]) of binding to WT ZIKV prM/E. At least two replicate values were obtained for each experiment.

### Antibody genetics of ZIKV type-specific and potently neutralizing human MAbs.

The human MAb A9E is a ZIKV type-specific and strongly neutralizing antibody that binds to a distinct epitope centered on E glycoprotein domains I and III. MAb B11F was isolated from a different individual and has an epitope that partially overlaps with the A9E epitope. Both of these MAbs are IgG1 antibodies with a lambda light chain (Table 2). In contrast to eight other MBC-derived monoclonal antibodies that neutralize Zika virus and have known V-D-J gene usage, B11F uses the V5-10 heavy-chain gene locus (11, 17, 19, 23–27). Heavy-chain V-gene usage also differs between B11F and A9E (Table 2). On the other hand, the B11F light-chain gene locus V2-14*01 is also used by at least three other Zika virus-neutralizing antibodies: A9E, G9E, and C10 (11, 26). All four of these monoclonal antibodies share similar CDRL3 (complementarity-determining region 3 of the light chain) sequences. Notably, B11F has fewer nonsilent somatic hypermutations (SHM) than A9E in both the V and H genes, suggesting a higher degree of somatic hypermutation, which may explain the stronger neutralization potency of A9E than of B11F despite similar E glycoprotein epitopes (Table 2). Stronger neutralization potency may suggest that MAb A9E has higher affinity for ZIKV E glycoprotein dimers than MAb B11F on a single-molecule level, and we plan to test this hypothesis in the future. Despite their different origins, MAbs B11F and A9E have similar CDRH3 and CDRL3 sequences, a finding consistent with their binding to a shared epitope on the E glycoprotein.

**TABLE 2:** Comparison of sequences and IgG characteristics of Zika virus monoclonal antibodies A9E and B11F^*a*^

Clone	Isotype	Heavy chain								Light chain						
		Gene usage			CDRH1, -2, -3 lengths (aa)	No. of nonsilent SHM	Ratio of nonsilent to silent SHM		CDRH3 sequence	Gene usage		CDRL1, -2, -3 lengths (aa)	No. of nonsilent SHM	Ratio of nonsilent to silent SHM		CDRL3 sequence
		V	D	J			FR^*b*^	CDR		V	J			FR	CDR	
B11F	IgG1(λ)	V5-10-1*03	D3-9*01	J6*03	8, 8, 20	5	2	0	AISLYYDISTGDNYYWYMDV	V2-14*01	J2*01, J3*01	9, 3, 11	6	1.5	3	SSYRSGSTLGV
A9E	IgG1(λ)	V3-23*01	D3-3*01	J6*03	8, 8, 17	23	3.25	10	ARSDFWRSGRYYYYMDV	V2-14*01	J2*01	9, 3, 11	11	0.86	4	SSYSISSTLLV

a Data for B11F are from https://www.ncbi.nlm.nih.gov/igblast/. A9E data are from the work of Collins et al. (11).

b FR, framework region.

## DISCUSSION

In this study, we have identified a new region on the ZIKV envelope glycoprotein targeted by two strongly neutralizing MBC-derived human monoclonal antibodies isolated from two separate individuals. Both MAbs bound to the monomeric form of the E glycoprotein, indicating that most of the footprint is contained within a single E protein molecule. In this regard, both antibodies are different from other human MAbs that strongly neutralize flaviviruses and bind to quaternary epitopes that span two or more E molecules on the viral surface (11, 12, 19).

By passaging ZIKV in the presence of MAb B11F to select for escape mutations, we identified a residue buried within the core of EDIII (M345) that was critical for binding and neutralization. We hypothesize that this mutation results in allosteric changes within EDIII that influence the surface epitope on EDIII or EDI that interacts with B11F during virus binding and neutralization. The A9E monoclonal antibody showed decreased (though still potent) binding and neutralization of the B11F escape mutant. Similarly, mutations in the EDI–EDIII hinge region that promoted escape from neutralization by A9E had a significant impact on B11F binding as well as neutralization. These observations indicate that although the footprints of these two monoclonal antibodies are similar and likely overlap, they are not identical. Binding and viral escape mutation studies, though good surrogates for predicting the viral epitopes of potent MAbs, are limited here in that they do not give us direct structural or functional data, which will have to be obtained in future studies in order to fully understand the epitopes and mechanisms of these MAbs.

While B11F did not bind to EDI alone, we predict that the footprint of this antibody is contained mainly in EDI, because of the many mutations in this domain identified by alanine-scanning mutagenesis (Fig. 4). Similarly, the majority of the epitope for A9E appears to be centered on EDI, because the antibody was able to bind to EDI alone produced as a recombinant antigen. While viral mutations in EDIII (B11F) or the linker region between EDI and EDIII (A9E) resulted in complete neutralization escape, neither MAb bound to EDIII when it was separated from the rest of the E glycoprotein. This indicates that both antibodies require interaction with EDI and an adjacent EDIII region for functional neutralization of ZIKV. However, the expanded footprints of the two antibodies differ, because EDIII mutations had distinct phenotypes for each MAb. Residues identified by alanine scanning as important are thought to be those most energetically important for antibody binding (28, 29). Although we identified residues for B11F only in EDI, it is possible that EDIII has epitope contact residues that, while not energetically important for binding, are subject to perturbation by escape mutation. Furthermore, the three alanine-scanning mutations with the highest energetic importance for B11F binding had little effect on A9E binding. Taken together, the mutagenesis and escape mutant studies reveal that B11F and A9E rely on different points of contact on the ZIKV E glycoprotein surface, with A9E covering the outer portion of the EDI–EDIII hinge and B11F shifted inward, covering EDI, with possible contacts on EDIII and EDII as well.

Previous studies from our group and other groups indicate that quaternary-structure epitopes centered on ZIKV EDII with footprints that expand into adjacent molecules of E glycoprotein homodimers and higher-order structures act as targets of strongly neutralizing and protective human antibodies (11, 12, 19). In this study, we propose that the A9E and B11F monoclonal antibodies define a new antigenic region spanning EDI and EDIII within a single E protein targeted by the neutralizing human antibody response to ZIKV. The EDI–EDIII interface is an important immunogenic epitope on the ZIKV E glycoprotein that can be leveraged for ZIKV vaccine design. Furthermore, both A9E and B11F have the potential to be used as future therapeutic antibodies for the treatment of ZIKV infection or as prophylaxis during a ZIKV outbreak.

## MATERIALS AND METHODS

### Human subjects and biospecimen collection.

Whole-blood donations were obtained from fully consenting volunteer travelers with self-reported risks for arboviral infection through the UNC Arboviral Traveler Study (IRB no. 08-0895). Plasma was isolated from whole blood by centrifugation and was analyzed by virus capture ELISA for binding to ZIKV and DENV-1 to -4. If antibody binding to any virus was observed, the neutralization titer was determined by a FRNT_50_ assay. Plasma samples with neutralization titers for one DENV serotype or for ZIKV that were 4-fold higher than all other titers were characterized as indicating a primary infection (11). Previously characterized flavivirus-positive serum samples were used as controls for ELISA and neutralization experiments.

### Viruses and cells.

ZIKV strain H/PF/2013 was obtained from the U.S. Centers for Disease Control and Prevention and was used in all assays (30). DENV WHO reference strains DENV-1 West Pac 74, DENV-2 S16803, DENV-3 CH54389, and DENV-4 TVP-376 were initially obtained from Robert Putnak (Walter Reed Army Institute of Research, Silver Spring, MD, USA). A9E escape mutant viruses were isolated as described previously (11). For cell culture-based experiments and the maintenance of virus stocks, Vero (Cercopithecus aethiops) cells (ATCC CCL-81) were used. Vero cells were grown at 37°C under 5% CO_2_ in Dulbecco’s modified Eagle medium (DMEM) supplemented with 5% fetal bovine plasma and l-glutamine. Virus stocks were titrated on Vero cells by a plaque assay or focus-forming assay. All studies were conducted under biosafety level 2 containment.

### Memory B-cell immortalization and sorting.

The B11F monoclonal antibody was generated from donor DT172 by using the 6XL method of memory B-cell immortalization (31). Briefly, peripheral blood mononuclear cells (PBMCs) from donor DT172 underwent CD22^+^ magnetic purification followed by flow cytometric sorting for CD19^+^ CD27^+^ IgM^−^ class-switched MBCs. These sorted MBCs were then transduced with the 6XL retrovirus and were activated by incubation with CD40L-expressing cells as well as human interleukin 21 (IL-21) to support antibody secretion and B-cell proliferation (32). 6XL-transduced MBCs then underwent flow cytometric sorting by green fluorescent protein (GFP) expression (as a marker of transduction) into polyclonal cultures at 50 GFP^+^ cells per well on a 96-well plate using a BD FACSAria III flow cytometer.

### Memory B-cell screening and monoclonal antibody generation.

Polyclonal MBC cultures were screened by ELISA for binding to both DENV-1 to -4 and ZIKV. Cultures that were ZIKV positive were single-cell sorted on a BD FACSAria III flow cytometer, grown on CD40L and IL-21, and then screened as described above after 4 weeks. ZIKV antibody-positive monoclonal cultures were further screened by FRNT_50_ assays in Vero cells at 1:2 and 1:8 dilutions. Positive monoclonal cultures underwent RNA isolation and nested PCR for human Ig(H) and Ig(L) genes, followed by sequencing using previously described primers (33). Sequences were analyzed by IgBLAST (https://www.ncbi.nlm.nih.gov/igblast/) and compared to germ line sequences to determine V_H_ and V_L_ gene usage, V(D)J gene usage, CDR3 sequences, rates of somatic hypermutation, and IgG isotypes. The complete heavy-chain and light-chain V region sequences were then cloned into IgG1 (GenBank accession no. FJ475055) and Ig(λ) expression vectors (GenBank accession no. FJ517647), respectively.

Heavy- and light-chain vectors were verified by sequencing and transformed into DH5α cells (New England BioLabs [NEB]). The transformed cells were grown, and the plasmid was purified with a Midi prep kit (Macherey-Nagel). The purified plasmid DNA (both heavy and light chain) was transfected into a 30-ml culture of HEK Expi293F cells (Thermo Fisher Scientific). The culture was harvested after 5 days, and the supernatant was affinity purified with preequilibrated MabSelect SuRe resin in a gravity column. The column was washed with 1× phosphate-buffered saline (PBS) and was eluted with 300 mM sodium citrate (pH 3.0) into six 475-μl fraction tubes containing 25 μl of 1 M Tris (pH 8.0).

### Capture ELISA.

Monoclonal antibody binding to ZIKV, DENV, and subunit envelope antigens was determined by a capture ELISA. A 96-well plate was coated with the murine monoclonal antibody 4G2 (UNC Center for Structural Biology) for ZIKV and DENV antigens, with an anti-mannose binding protein (anti-MBP) monoclonal antibody (ProteinTech) for Zika virus EDI and EDIII antigens (34), or with anti-His (Invitrogen) for the ZVrecE80 antigen in 0.1 M carbonate buffer (pH 9.6). Plates were coated for 1 h at 37°C and were then washed with 1× Tris-buffered saline (TBS) plus 0.2% Tween buffer using a plate washer (BioTek). Three percent nonfat milk (in 1× TBS plus 0.05% Tween buffer) was used to block the plate. Antigens were added as follows: ZIKV diluted 1:1, EDI (200 ng per well), EDIII (200 ng per well), ZVrecE80 (500 ng per well). Antigens were diluted in blocking buffer, and plates were incubated for 1 h at 37°C and then washed as described above. B11F and control monoclonal antibodies were added to the plate at 100 ng per well. We used alkaline phosphatase-conjugated goat anti-human IgG (Sigma) diluted 1:2,500 in blocking buffer as a secondary antibody. Each incubation step was carried out for 1 h at 37°C. *p*-Nitrophenyl phosphate (PNPP) (Sigma) was added as a substrate to develop the plate, and absorbance at 405 nm was measured using a plate reader (BioTek). All ELISA experiments were carried out in duplicate, as at least three independent experiments.

### Neutralization assay.

Neutralization titers were determined by a 96-well microFRNT assay as described previously (11). Briefly, serial dilutions (1:4) of a monoclonal antibody were mixed with 50 to 100 focus-forming units of virus in 2% fetal bovine serum (FBS)–DMEM. The virus-antibody mixtures were incubated for 1 h at 37°C and were then transferred to a monolayer of Vero cells for infection for 40 h with ZIKV (H/PF/2013). Cells were then fixed and permeabilized. Infected cells were stained with primary antibodies 4G2 (HB-114; ATCC) and 2H2 (UNC Center for Structural Biology) for 1 h at 37°C, washed, and then incubated with a horseradish peroxidase-conjugated goat anti-mouse secondary antibody (KPL) for 1 h at 37°C. Foci were visualized with 50 μl of TrueBlue (KPL) and were counted using a CTL enzyme-linked immunosorbent spot (ELISpot) reader. Cell-only controls and ZIKV-positive cell controls were also added to each plate. Neutralization experiments were carried out in duplicate, as at least three independent experiments.

### BOB assay.

BOB was performed as described previously (11). Briefly, a 96-well plate was coated with 4G2 at 100 ng/well, and the plate was blocked with 3% nonfat milk diluted in 1× TBS plus 0.05% Tween. ZIKV was diluted 1:1 in blocking buffer and was added to the plate. Monoclonal antibodies were serially diluted 1:4 in blocking buffer and were added to the plate starting at 100 ng/well. B11F was conjugated with alkaline phosphatase (Abcam) and was added to the plate at 100 ng/well. The PNPP substrate (Sigma) was added, and the absorbance at 405 nm was measured (BioTek).

### Escape mutant selection and sequencing.

ZIKV (multiplicity of infection [MOI], 0.01) was incubated with different multiples of the FRNT_50_ of B11F for 1 h at 37°C. The virus–monoclonal antibody mixture was added (2 ml) to Vero cells in a 6-well plate (Greiner). After the cells were infected for 1 h at 37°C, the supernatant was discarded, and 1 ml 2% fetal calf serum (FCS) medium (Gibco) plus 1 ml B11F diluted in 2% FCS was added to the plate. Wild-type ZIKV was passaged as a control in medium alone alongside virus undergoing B11F selection, along with a cell-only control. Aliquots (150 μl) were taken at a 3-h baseline and at 24, 48, and 72 h after infection for quantitative reverse transcription-PCR (RT-PCR), and cytopathic effects were observed under a microscope at each time point. Three days after infection, 1 ml of the supernatant was passaged to a new plate of Vero cells plus 1 ml 2% FCS medium. RNA was isolated from the cell culture supernatants and was converted to cDNA (NEB). The E genes of the stock virus, passaged virus control, and passaged virus plus B11F were sequenced via RT-PCR. The PCR product was run on a 2% agarose gel, gel extracted, and purified (Zymogen). The purified DNA product was submitted for sequencing. The ZIKV stock, passaged control, and passaged virus plus B11F were aligned via SnapGene. Mutations were observed and presented using PyMOL.

### Alanine-scanning mutagenesis.

Alanine-scanning mutagenesis was carried out by Integral Molecular on an expression construct for ZIKV prM/E (strain ZikaSPH2015; UniProt accession no. Q05320). Residues were mutagenized to create a library of clones, each with an individual point mutant (13). Residues were changed to alanine (with alanine residues changed to serine). The resulting ZIKV prM/E alanine scan library covered 100% of target residues (672 of 672). Each mutation was confirmed by DNA sequencing, and clones were arrayed into 384-well plates, one mutant per well.

Cells expressing ZIKV E mutants were immunostained with B11F MAb, and mean cellular fluorescence was detected using an Intellicyt flow cytometer. Mutations within critical clones were identified as critical to the monoclonal antibody epitope if they did not support the reactivity of the MAb but did support the reactivity of other conformation-dependent monoclonal antibodies (Fig. 4C). This counterscreen strategy facilitates the exclusion of Env mutants that are globally or locally misfolded or that have an expression defect (35). Validated critical residues represent amino acids whose side chains make the highest energetic contributions to the monoclonal antibody–epitope interaction (28, 29).
